# A Study on the Electrochemical Performance of SLMed Al6061/TiB2 Composite Anodes Caused by Laser Power

**DOI:** 10.3390/molecules30051183

**Published:** 2025-03-06

**Authors:** Jitai Han, Kui Zhu, Chenglong Li, Yin Li, Sida Tang, Peng Li

**Affiliations:** 1 School of Automation, Wuxi University, Wuxi 214105, China; 860085@cwxu.edu.cn (J.H.); 202312490366@nuist.edu.cn (Y.L.); 860219@cwxu.edu.cn (S.T.); 2 School of Automation, Nanjing University of Information Science and Technology, Nanjing 210044, China; 3 School of Mechanical Engineering, Jiangnan University, Wuxi 214000, China

**Keywords:** aluminum–air battery, Al6061/TiB2 composite anodes, SLM, laser power

## Abstract

Aluminum–air batteries have attracted more attention in recent years due to the theoretical possibility of replacing lithium batteries. Al6061/0.5wt.%TiB_2_ is considered a suitable anode material due to decreased hydrogenation corrosion. In this work, laser power was optimized via a selective laser melting process to increase the electrochemical and discharge performance of an Al composite anode. Relative density was studied in this work, and the formation mechanism caused by molten pool morphology was also researched using finite element analysis and experiments. The self-corrosion rate, open-circuit potential, polarization curve, EIS curve, and constant-current discharge performance were all studied in the following section, and the relationship between anode quality and laser power was discussed accordingly. The testing results revealed that when laser power reached 340 W, the Al6061/0.5wt.%TiB_2_ composite anode reached a relative optimal condition as defects reduced to a minimum value at this point, which resulted in overall anode performance increasing in the electrochemical and discharge test.

## 1. Introduction

**Figure 1:**
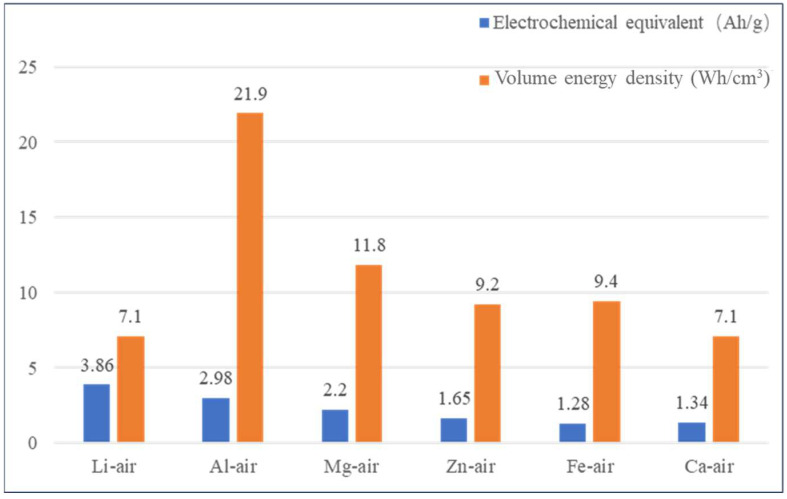
Comparison of energy density of different metal–air batteries.

With the global progress of science and technology and the gradual increase in the level of industrialization, the demand for energy for human activities is increasing, and the environmental problems caused by the excessive consumption of petrochemical resources are becoming more and more prominent [1,2]. The aluminum–air battery (AAB) is now attracting more and more attention due to some obvious advantages, such as high-current discharge, high specific energy, cheap and easy-to-obtain electrode materials, and so on, which is considered as “Green energy for the 21st century” [3]. However, due to the hydrogenation corrosion of the Al anode in alkaline electrolytes, the AAB’s life is seriously limited. As shown in Figure 1, the following is a comparison of the energy density of different metal-air batteries.

To improve grain refinement and organization, TiB_2_ was included as an improvement phase [4,5,6]. Traditional manufacturing methods like squeeze casting do not have high enough processing temperatures, which can cause compositional segregation of composites, larger grains, and uneven microstructure distribution, affecting molding quality [7]. To bind metal powder, additive manufacturing technology selective laser melting (SLM) was used to melt it. This method directly forms nearly fully dense metal parts with good mechanical properties without material agglomeration, gas inclusions, interfacial microcracks, or microstructural inhomogeneity, unlike traditional casting and powder metallurgy [8]. Dongdong Gu et al. [9] created single-pass aluminum alloy samples by SLM and examined the impact of the rich heat buildup in the formed layer and the rough forming surface on single-pass wetting. The selection strategy of scanning spacing and profile parameters greatly impacts the organization and mechanical characteristics of AlSi12 components manufactured by SLM, according to Prashanth et al. [10]. The results demonstrated that contour parameters reduce component ductility owing to surface residual stresses. This study did not examine how SLM parameters affected component microstructure. Li et al. [11] discovered that the high cooling rates of AlSi10Mg during SLM caused crystals. With the selected SLM settings, Akram et al. [12] computed grain structure evolution for multilayer deposition. The results showed that process parameters control grain size and orientation, and cooling rates impact alloy microstructure. Si and Mg in aluminum alloys can modify microstructure and mechanical characteristics during SLM processing; hence, anodic aluminum alloy models should be chosen carefully. Aluminum alloys like Al6061 solidify and liquefy fracture due to a high CTE (coefficient of thermal expansion). The lower thermal expansion of AlSi10Mg compared to Al6061 makes it a preferred SLM alloy. By preheating the substrate at 500 °C with precise process conditions, Uddin et al. [13] created crack-free components. Samples made without substrate preheating were unsatisfactory. Reinforcing phase nanoparticles were tested on Al6061 and Al7075 alloy powders by Martin [14]. In the generated portions, reinforcing phases regulated solidification and minimized internal cracks. Round pores lowered mechanical properties relative to as-built alloys. The literature has not examined how SLM process parameters affect thermal fracture development in Al6061-formed components.

This study examines AAB performance using Al6061/TiB_2_ as an anode. Laser power is tuned to increase anode printing quality, which improves AAB properties.

## 2. Results and Discussion

### 2.1. Relative Density

**Figure 2:**
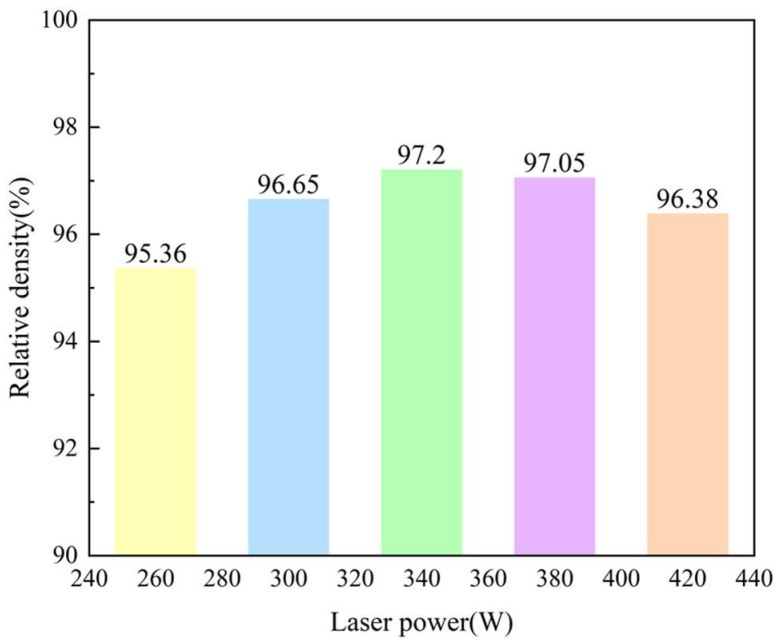
Relative density change in the samples printed with the different laser powers.

The density of the printed samples was measured using the Drainage Method, which can be seen in Figure 2 It can be found that the value showed an increasing trend with the increasing of laser power from 260 W to 340 W while reduced from 97.20% to 96.38% with a further laser power increasing from 360 W to 420 W. To prove the results gained here, Optical Microscopy was employed to observe the defects in these samples, as shown in Figure 2.

**Figure 3:**
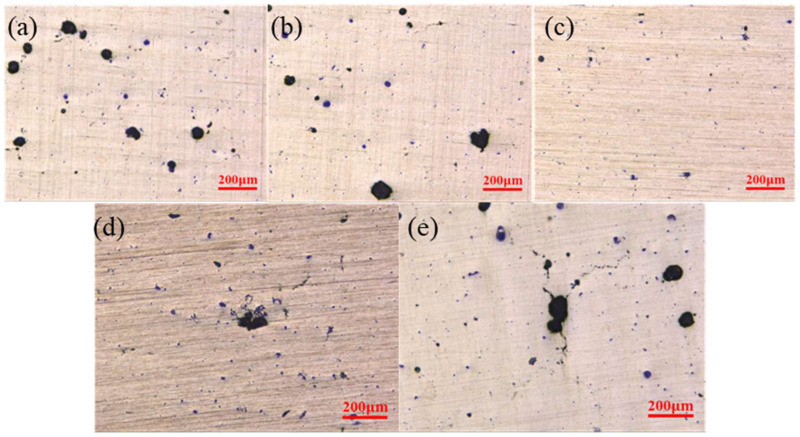
Defects shown on the samples printed using different laser powers. (**a**) 260 W; (**b**) 300 W; (**c**) 340 W; (**d**) 380 W; (**e**) 420 W.

Figure 3 above shows surface defects on samples printed with different laser powers.The size and number of the defects showed a quite significant reduction trend with the increasing of laser power to 340 W, and the defects became quite obvious with a further increasing of laser power to 420 w. This is because the laser power is too low, and the energy transfer from the laser heat source to the powder bed is limited during the forming process. The resulting molten pool has a lower temperature. The convective intensity of Malanguni decreased, and the viscosity of the liquid phase was higher. The presence of unmelted micron TiB_2_ particles will further increase the melt viscosity, liquid phase liquidity, and narrow shallow mo. The powder particles cannot be completely melted into the liquid phase metal, and the insufficient liquid phase will make it difficult to fully wet the scanning path and the previous solidification layer, resulting in insufficient fusion between adjacent paths and layers and defects like holes during solidification, reducing sample densification.

With laser power elevation, Marangoni convection in the molten pool increased, liquid phase viscosity decreased, and molten pool fluidity improved, which improved liquid aluminum wettability on TiB_2_, spreading property on TiB_2_ particles, and rearrangement process of TiB_2_. Due to the significant increase in alloy powder melting volume, sufficient liquid phase metal was produced, the melt pool was better wetted, larger, and had better metallurgical bonding between layers and melt channels.

**Figure 4:**
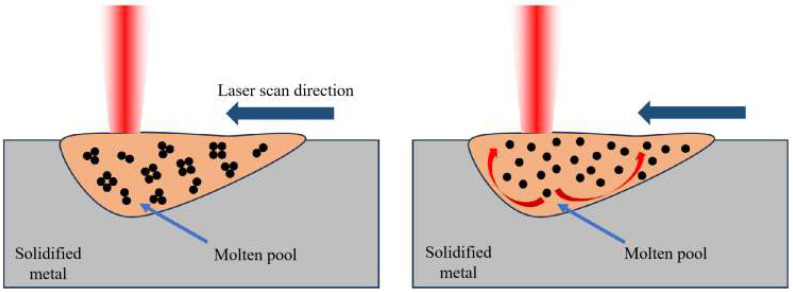
Schematic diagram of Marangoni convection in printing.

As the laser power increases, excessive energy is input into the melt pool, overheating it, pushing the melt pool center to the edge of the very high temperature gradient caused by a strong inward Marangoni flow, exacerbating the instability of the melt flow, increasing gas capture, and making porosity defects easy to form. Figure 4 below shows the schematic diagram of Marangoni convection. Shrinkage holes form between the matrix and reinforced phase. High-power laser energy also causes the melt pool to be too big, the liquid phase surface energy to be too low, and the melt pool stability to be compromised, causing interior metal droplets to splash violently. Spheroidization defects, which affect the flatness of the solidified layer surface, the difficulty of laying powder, and the interlayer’s metallurgical quality, are easily caused by splashing droplets that form micron-sized fine metal balls on the current layer. The melting pool temperature is too high, resulting in low melting points of Zn, Mg, and other elements, selective evaporation, and metal vapor. SLM’s high cooling rate prevents metal vapor from escaping, causing recoil pressure on the melting pool to form irregularly shaped locking hole defects. The aforementioned three factors reduce molded part density.

**Figure 5:**
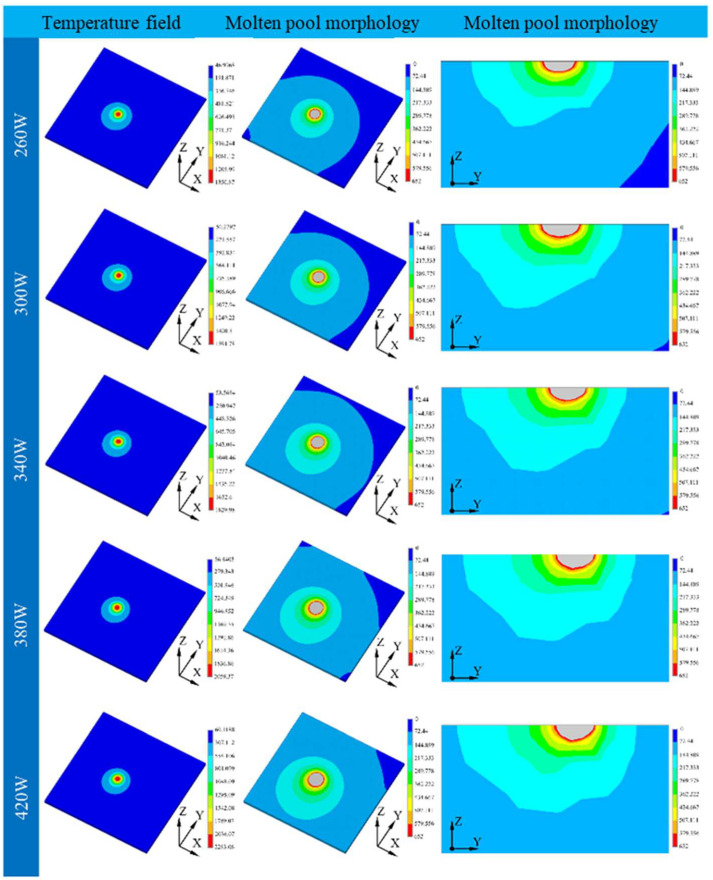
Cloud view of temperature field distribution for different laser powers.

To prove the theory proposed here, finite element theory was employed in this part to study the molten pool of the samples printed under different laser powers. The temperature distribution and morphology of the molten pool are shown in Figure 5.

**Figure 6:**
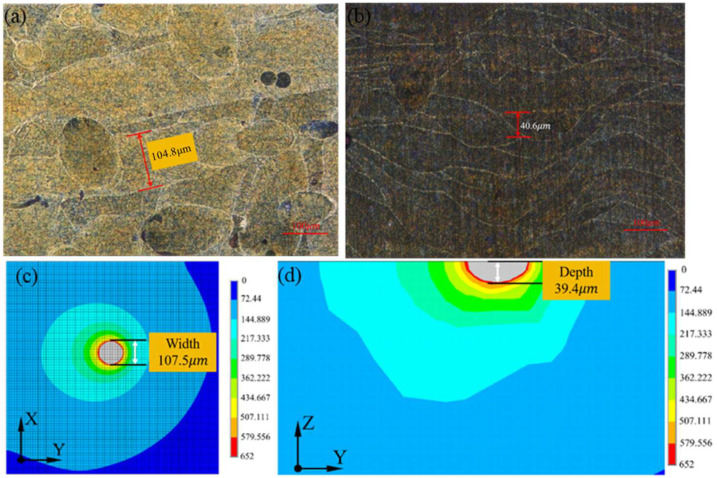
Comparison of molten pool width and depth of simulation and actual printing. (**a**) Actual molten pool width; (**b**) actual molten pool depth; (**c**) simulated molten pool width; (**d**) simulated molten pool depth.

To verify the validity of the simulation results, the sample printed using 340 W laser power was selected randomly and compared with the simulation results, as shown in Figure 6.

**Figure 7:**
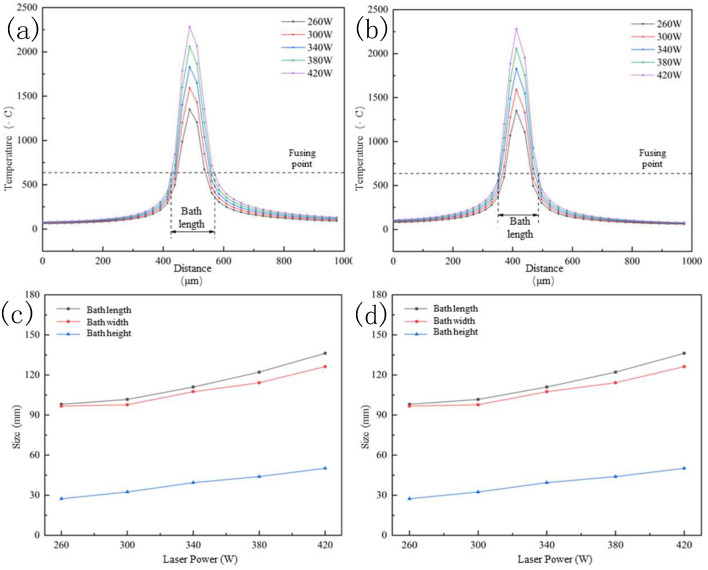
Molten pool size at different laser powers. (**a**,**b**)The bath temperature of different laser power; (**c**) length, width and height of the molten pool; (**d**) Aspect ratio of the molten pool.

It can be found that the molten pool width and depth were 107.5 μm and 39.4 μm, respectively, in the simulation, while it was 104.8 μm and 40.6 μm in the experiment. The error between the simulation and experiment was 2.6% and 3.0%, which confirmed the effectiveness of the simulation results. The molten pool size was calculated using the melting point of the materials, and the detailed sketch can be seen in Figure 7. The similar length/depth ratio of the molten pool printed under 340 W to 420 W further confirmed the theory presented before.

### 2.2. Self-Corrosion Rate

**Figure 8:**
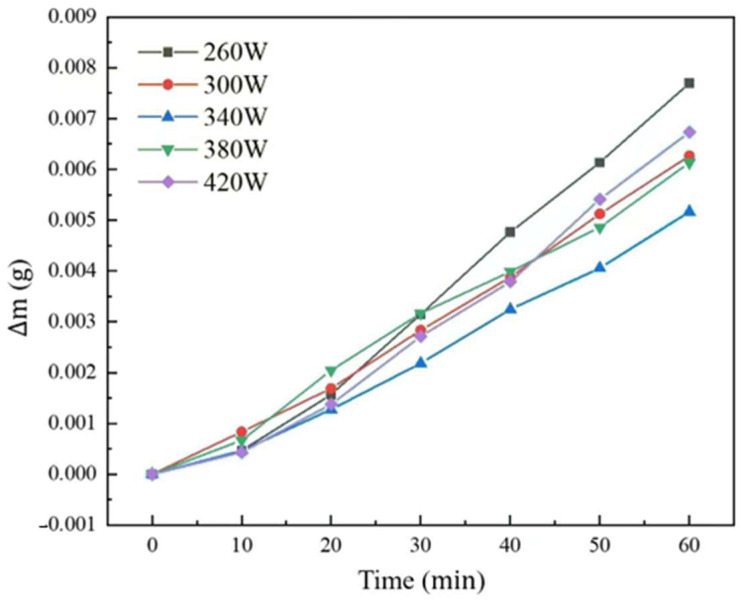
Weightlessness curve of the aluminum anode printed under different laser powers.

The weightlessness curve of the aluminum anode printed under different laser powers was shown in Figure 8, and linear fitting was applied to reveal the corrosion rate of each sample seen in Table 1.

It can be seen that with the increasing of laser power from 260 W to 420 W, the weight loss reduced from 1.344 × 10^−4^ g·cm^−2^·min^−1^ to 8.804 × 10^−5^ g·cm^−2^·min^−1^ and then increased to 1.163 × 10^−4^ g·cm^−2^·min^−1^. The first decreasing self-corrosion rate period was mainly caused by the easier electrolyte penetration due to the poor printing quality and more pore defects. As for the second increasing period, in addition to the reason given above, the large grain structure due to high laser power also leads to easy penetration of the electrolyte into the substrate, resulting in increased anode self-corrosion rates.

### 2.3. Open-Circuit Potential

**Figure 9:**
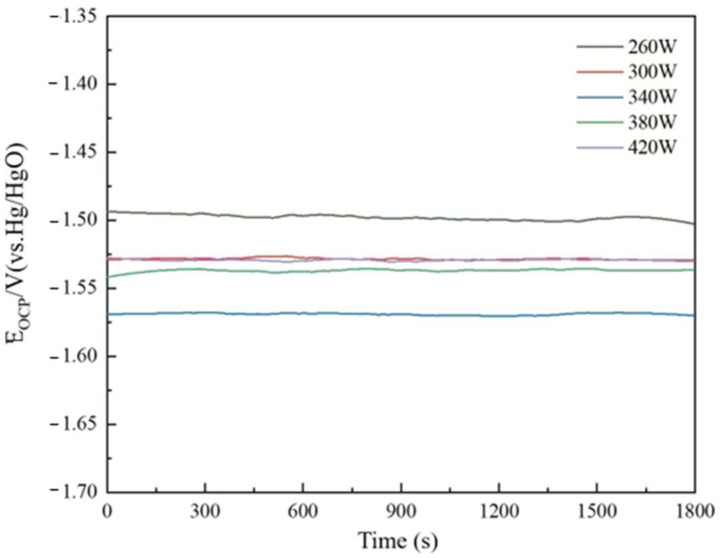
Open-circuit potential of the sample printed under different laser powers.

The open-circuit potential of the sample printed under different laser powers can be seen in Figure 9. The specific parameters are listed in Table 2. The material’s open-circuit potential is −1.499 V at 260 W laser power. As laser power increases to 340 W, the material’s open-circuit potential drops to −1.569 V. With increasing laser power, the powder bed temperature in the molding process rises, precipitating some less reactive trace elements and harmful impurities from the matrix to the anodic alloy surface, increasing the surface’s active point rapidly. Some hazardous contaminants can create corrosion microcells with a pure aluminum matrix, speeding sample surface response and lowering open-circuit potential. As laser power increases, the metal liquid phase stays too long, the sample grain experiences overgrowth, the number of grain boundaries decreases, reducing the discharge active sites of the anode alloy, and the open-circuit potential becomes positive. At 420 W, the material’s open-circuit potential is −1.529 V.

### 2.4. Polarization Curve

**Figure 10:**
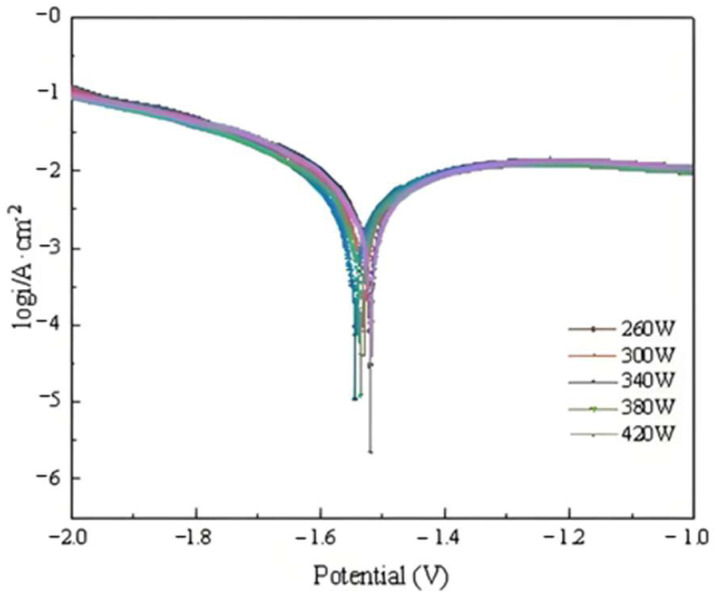
Polarization curve of the sample printed under different laser powers.

The polarization curve of the sample printed under different laser powers can be seen in Figure 10, and the parameters calculated using Tafel Extrapolation are shown in Table 3.

It can be seen that when the laser power gradually increases, the corrosion potential of aluminum anode samples at 260 W, 300 W, 340 W, 380 W, and 420 W is −1.520 V, −1.530 V, −1.544 V, −1.536 V, and −1.519 V, respectively, and the trend is constantly negative and then positive. The sample with the lowest polarization had the lowest corrosion potential at −1.544 V at 340 W laser power. The appropriate laser power can promote TiB_2_ distribution in the matrix, provide more nucleation sites, promote microcrystalline organization, and impede columnar crystal growth, resulting in a uniform microstructure distribution and uniform corrosion of the alloy. The larger grain boundary area provides more reaction sites and higher electrochemical activity.

According to the fitted electrochemical parameters, Icorr declined and then increased when the laser power was gradually lowered from 420 W. When the laser power was reduced from 420 W to 260 W, the aluminum anode samples exhibited corrosion current densities of 5.014 × 10^−3^ A/cm^2^, 4.823 × 10^−3^ A/cm^2^, 3.819 × 10^−3^ A/cm^2^, 4.467 × 10^−3^ A/cm^2^, and 4.692 × 10^−3^ A/cm^2^, along with polarization resistances of 9.2 Ω·cm^2^, 10.1 Ω·cm^2^, 12.4 Ω·cm^2^, 11.2 Ω·cm^2^, and 10.3 Ω·cm^2^, respectively. At 340 W laser power, the composite sample exhibited the lowest corrosion current density and the highest polarization resistance, indicating a low self-corrosive rate. The right laser power may fully melt the powder while eliminating flaws such as spattering and porosity, enhancing material forming quality, and decreasing uneven self-corrosion in pores.

### 2.5. EIS Curve

**Figure 11:**
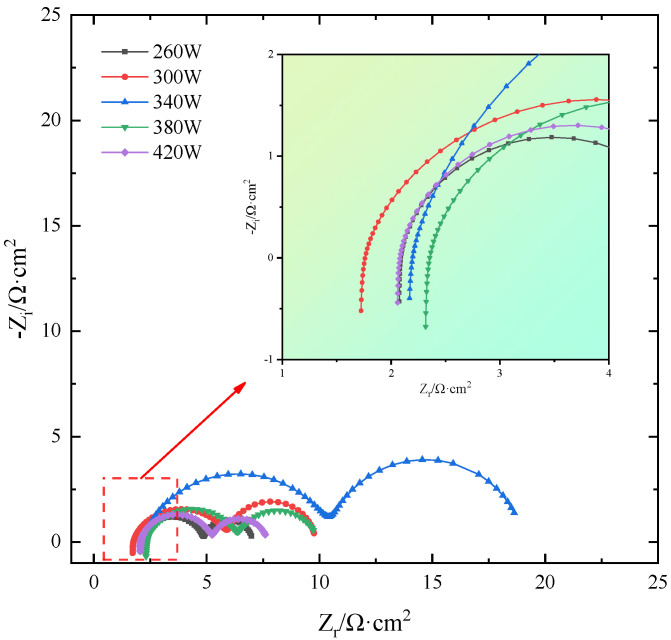
EIS fitting curves of SLM-formed TiB_2_/6061 composite anode.

The EIS fitting curves of SLM-formed TiB_2_/6061 composite samples in salt-based alkaline electrolytes under different laser powers are shown in Figure 11, and Table 4 shows the EIS AC impedance fitted parameters. The figure shows that as laser power increases, the size of the semicircular capacitance arcs in the sample’s EIS curves increases first and then decreases because the larger the radius, the better the samples’ corrosion resistance. Therefore, sample corrosion first increases and then decreases. Since the typical sample’s corrosion resistance improves with the capacitance arc radius, its corrosion degree reduces first and subsequently increases. The correlation coefficient χ^2^ in the fitting parameter table reflects the degree of correlation between the fitted data and test data for each anode alloy. A smaller number implies more accurate fitted data. Table 4 shows that the χ^2^ values of the fitted data for each sample are below 10^−3^. This suggests that the data more accurately reflect the results of EIS testing of the anode alloys in a salt-based alkaline electrolyte.

**Figure 12:**
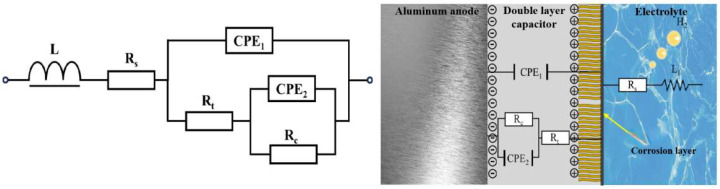
Equivalent circuit of TiB_2_/6061 anode in composite electrolyte system.

As seen in the table, when laser power increases, each sample’s Rt and Rc differ significantly and increase and subsequently decrease. On one side, this is due to the laser power being too high or low, or the laser input energy being too low or high, preventing powder fusion or melt pool stability. This phenomenon severely affects forming quality, resulting in more defects in the sample, electrolyte penetration into the sample interior, and inhomogeneous corrosion; on the other hand, a low molten pool temperature will cause the organization to be not uniform enough during crystallization generation, and a high pool temperature will produce grains. Fusion and annexation generate coarse grains, which increase electrolyte erosion depth and reduce corrosion resistance. Figure 12 shows the schematic diagram of the equivalent circuit. The sample exhibits better corrosion resistance when the laser power is 340 W, as the inductive resistance triggered by hydrogen precipitation is small (7.316 × 10^−7^ H·cm^2^) and the R_t_ and R_c_ are large (8.504 Ω·cm^2^ and 8.348 Ω·cm^2^, respectively), consistent with self-corrosion experiments and polarization curve analysis.

### 2.6. Constant-Current Discharge Performance

**Figure 13:**
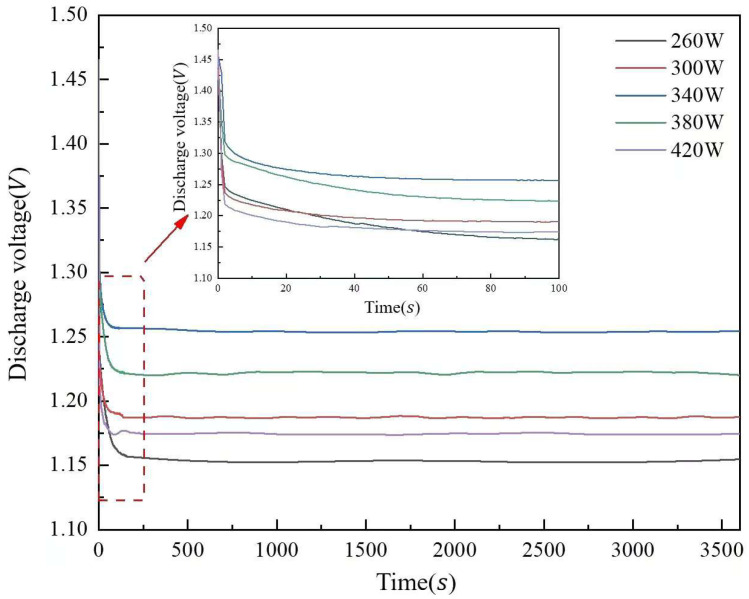
Discharge voltage curves of aluminum anode printed under different laser powers.

Figure 13 illustrates the discharge voltage curves of aluminum anode printed under different laser powers. The discharge voltage of the aluminum–air cell is 1.153 V at 260 W laser power, 2.95% and 8.76% higher at 300 W and 340 W, respectively, and 1.254 V at 340 W. When the laser power is raised to 380 W and 420 W, the discharge voltage drops 2.55 and 6.38% from 1.254 V. When the laser power is raised to 380 W and 420 W, the discharge voltage drops 2.55 and 6.38% from 1.254 V. The surface pore distribution, densification, and microstructure morphology of the aluminum matrix composite anode cause the discharge voltage to increase and then decrease with laser power. The reason for this phenomenon is related to the improvement of the anode surface quality. As the anode surface defects decrease, the contact area between the anode and the electrolyte increases, which improves the discharge performance. When the laser power reaches 340 W, the surface quality of the anode is optimized with a more uniform pore distribution and better material densification, resulting in the highest discharge voltage.

However, when the laser power exceeds 340 W, the discharge voltage starts to decrease. This may be due to the excessive laser energy, which leads to an increase in pore size and an increase in surface defects. These defects (e.g., deep grooves and corrosion pits) increase electrolyte penetration and hydrogen precipitation, which reduces the discharge voltage and overall cell performance.

**Figure 14:**
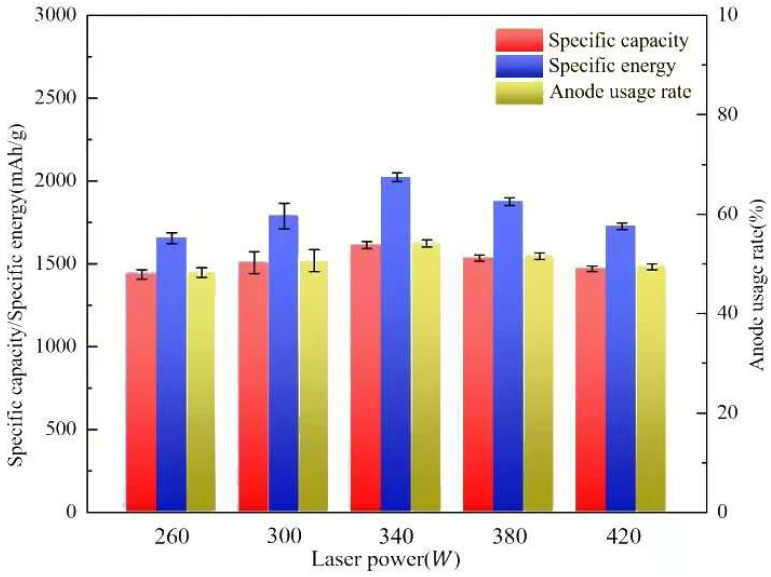
Specific capacity, specific energy, and usage rate of the anode printed under different laser powers.

Table 5 gives discharge performance data for aluminum–air batteries, and Figure 14 illustrates changes in the specific capacity, specific energy, and anode usage rate under different laser powers. The anode utilization rate increases and then decreases with laser power. At 260 W, it is 48.25%, increases by 5.10% and 12.25% at 340 W and 420 W, respectively, and reaches its maximum of 54.16% at 340 W. When the laser energy density is increased to 300 W and 340 W, the anode utilization rate increases by 5.10% and 12.25%, respectively, reaching 54.16% at 340 W. When the laser power is increased to 380 W and 420 W, it decreases by 4.86% and 8.81%, respectively. The aluminum–air battery has 1436.15 mA/g at 260 W laser energy density, similar to the anode use. The specific capacity rose by 4.95, 12.28, 6.83, and 2.40% with laser power, demonstrating that it increases and then drops. In addition, the discharge voltage and specific capacity were plotted to show the evolution of the aluminum–air battery’s specific energy with laser energy density. At 260 W, the specific energy was 1656.52 Wh/kg, enhanced laser power to 300 W and 340 W, and enhanced specific energy by 8.22% and 22.08%, respectively. Increasing laser energy density to 380 W and 420 W improved specific energy by 8.22% and 22.08%. The aluminum–air battery’s specific energy increased by 4.95, 12.28, and 6.83 percent. At 380 W and 420 W, the specific energies were 1875.23 Wh/kg and 1727.12 Wh/kg, respectively. These values were reduced by 7.27% and 14.59% compared to the aluminum anode at 340 W laser power. Specific energies also rose and decreased with laser power.

**Figure 15:**
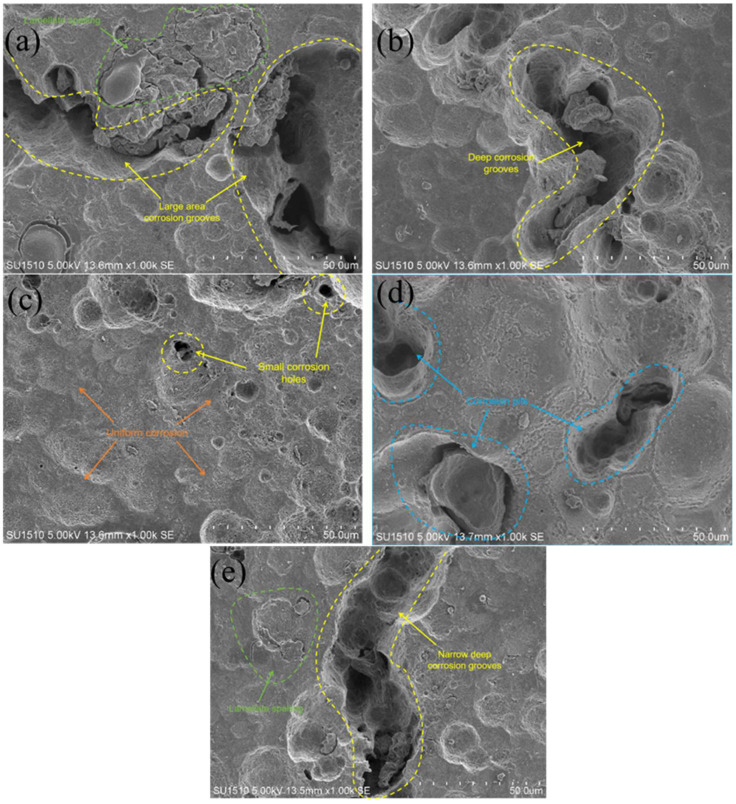
Surface morphology of anodes after discharge printed under different laser powers. (**a**) 260 W; (**b**) 300 W; (**c**) 340 W; (**d**) 380 W; (**e**) 420 W.

**Figure 16:**
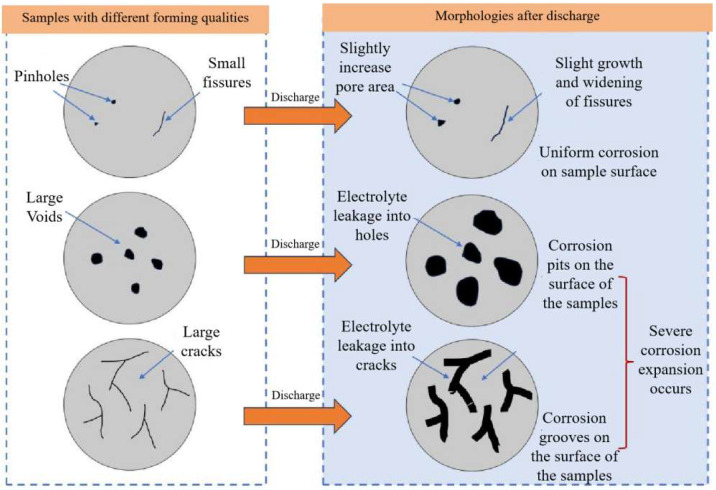
Schematic diagram of defects formation under different anode initial state.

Figure 13 and Figure 14 show that in the moderate laser power interval, the discharge voltage, anode utilization, specific capacity, and specific energy of the aluminum–air batteries increased and then decreased due to the surface pore distribution, densification, and microstructure morphology of the porous aluminum anode. SEM was used to determine the surface morphology of the aluminum matrix composite anode after discharge to assess how laser power affects porous aluminum anode discharge performance (Figure 15). The figure shows that the discharge surface of 260 W and 300 W aluminum matrix composite anodes is rough and has corrosion grooves with lengths exceeding 50 μm, indicating severe corrosion. When the laser power is 340 W, the discharge surface of the aluminum composite anode is relatively flat, the corrosion morphology shows obvious and crystalline corrosion, the aluminum anode in the discharge process shows uniform dissolution characteristics, the discharge process is stable, the number of active sites of the self-corrosion reaction is small, and hydrogen precipitation has been suppressed, so the aluminum anode alloy under the power As laser power increases to 380 W and 420 W and the corrosion degree of the aluminum anode worsens, resulting in larger pits and deeper grooves with a diameter of 38 μm. This is linked to poor composite material forming quality. Figure 16 shows that when the aluminum anode sample has fewer pore defects and smaller sizes, surface tension inhibits the electrolyte from penetrating the defects. As a result, discharge dissolution and hydrogen corrosion are more uniform, and the corrosion morphology is relatively flat. When the material has many cracks or holes, the electrolyte penetrates to increase the corrosion area and discharges to form corrosion pits and grooves, allowing more aluminum to participate in the hydrogen precipitation corrosion reaction. In conclusion, aluminum composite anodes consume anode material for both discharge and hydrogen precipitation reactions, but the sample with high forming quality has a low hydrogen precipitation corrosion rate, so the anode mostly undergoes discharge and has high discharge performance.

## 3. Methods

**Figure 17:**
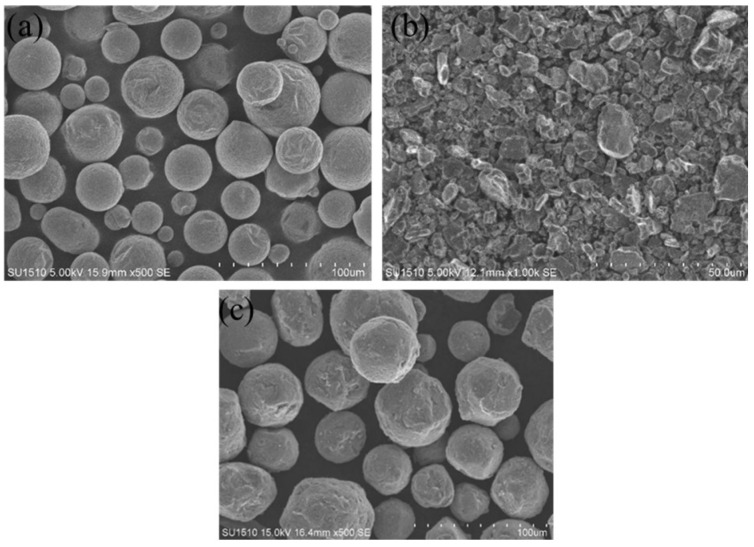
TiB2/6061 powder microscopic morphology and physical picture of ball milling device. (**a**) 6061 powder; (**b**) TiB_2_ powder; (**c**) 0.5 wt.% TiB_2_6061.

This study utilized Al6061 powder from Grinm Additive Manufacturing Technology Co., Ltd., with particle sizes ranging from 20 to 63 μm. Chemical composition of the powder is shown in Table 6. GRIPM Advanced Materials Co., Ltd. (Suzhou, Jiangsu Province, China). supplied TiB2 reinforcement. Figure 17 shows the morphology of 6061, TiB2, and combined 6061+ 0.5wt.% TiB2. Nanjing Nanda Instrument Co., Ltd. (Nanjing, Jiangsu, China) supplied this planetary ball mill. With 2.5:1 ball-to-material ratio, this piece rotated at 275 rmp for 2 h. It rotated 15 min in one direction and 5 min in the other to increase dispersion uniformity. To enhance print quality, the powder was oven-dried at 100 °C for 5 h. This provides a good dry environment for powder grinding.

**Figure 18:**
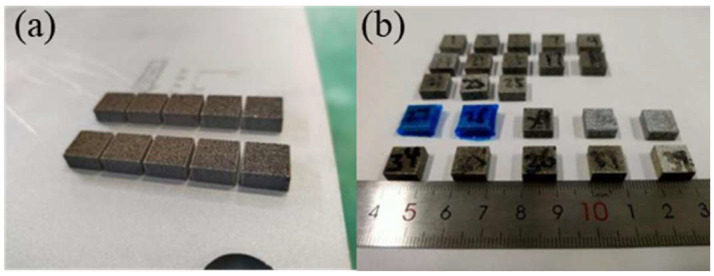
SLM-formed aluminum matrix composite samples. (**a**) Samples on base plate; (**b**) samples after wire cutting.

The XDM250 (Suzhou, Jiangsu, China) under-feed laser selective zone melting equipment from Xidimo 3D Printing Technology was used to test TiB_2_/6061 composites for SLM formation. Figure 18 shows the equipment’s appearance, and Table 7 lists its parameters. Since aluminum and titanium have a strong affinity for oxygen, oxide impurities will form in the SLM high-temperature forming test environment, affecting forming quality [15], so the equipment uses argon to fill the forming compartment as a protective gas to keep the oxygen content below 100 ppm.

This research examines how laser power affects TiB_2_/6061 composite molding quality. Densification, corrosion resistance, and discharge qualities were studied accordingly. Layer thickness and spot size were maintained at 30 μm and 50 μm, respectively, throughout the experiment. Unidirectional linear scanning with 67° layer rotation was used. The experimental program is in Table 8.

**Figure 19:**
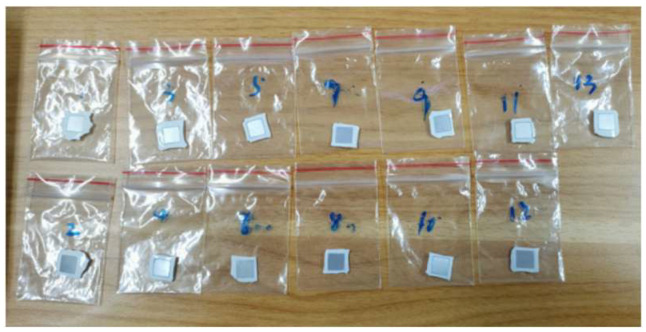
Self-corrosion test samples.

3 the sample from the substrate after the SLM forming test. The formed samples are depicted in Figure 19 YMP-2 grinding and polishing machine uses 400 mesh, 800 mesh, 1200 mesh, and 2000 mesh metallographic sandpaper and velvet polishing cloth on TiB_2_/6061 sample grinding and polishing treatment to avoid sample surface oil, burrs, and scratches on subsequent test results.

The samples’ corrosion resistance was assessed using self-corrosion and electrochemical tests. Self-corrosion samples were 1 cm × 1 cm × 0.1 cm TiB_2_/6061 aluminum matrix composites with 1.0 cm^2^ effective working surface area and additional coating of 704 organic silica gel for sealing. The weight loss method was used to test the samples’ self-corrosion rate. After 1 h in a mixed electrolyte of 0.3 mol/L NaOH and 4 mol/L NaCl at room temperature, the samples were removed and placed in dilute nitric acid solution for 120 s at 10 min intervals to dissolve corrosion products from the aluminum surface.

Subsequent ultrasonic cleaning with anhydrous ethanol removes the surface and the corrosion products remaining in the corrosion pits, which are dried with a hair dryer and then weighed and recorded, and the average of the change in mass before and after corrosion is denoted as ∆*m*, with a value accurate to 0.001 g. The rate of self-corrosion is calculated according to Equation (1).

(1) $$\nu_{a}=\frac{\Delta m}{s\times t}$$

where *v_a_* was the self-corrosion rate, g·cm^−2^·min^−1^, Δ*m* was the anode weight loss, g, *s* was the effective working space, cm^2^, and *t* was the soaking time, min.

**Figure 20:**
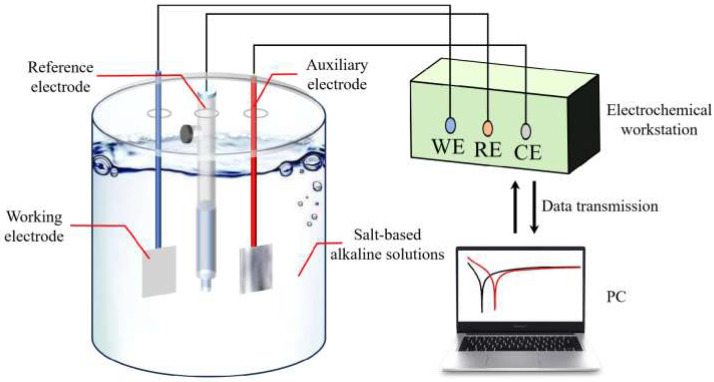
Schematic diagram of electrochemical test.

The CHI750E electrochemical workstation was used to test the electrochemical properties of the samples, and the experimental procedure is shown in Figure 20. A three-electrode system was used for the test, in which the reference electrode was a saturated calomel electrode (the potential of the reference electrode was 0.241 V), the auxiliary electrode was a 1 cm × 1 cm platinum (Pt) sheet electrode, the working electrode was treated in the same way as in the self-corrosion test, and the whole test was carried out at a temperature of 25 ± 1 °C.

**Figure 21:**
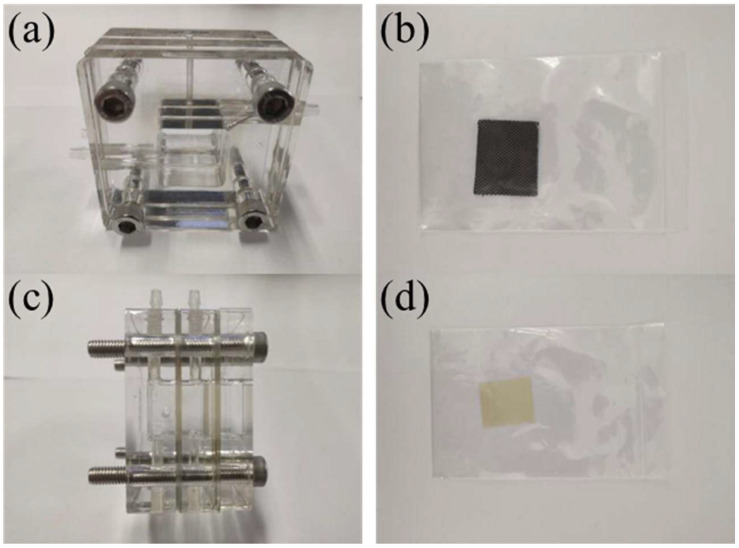
Aluminum-air battery discharge tester. (**a**) Front view of double electrolyte mold; (**b**) cathode carbon paper; (**c**) side view of double electrolyte mold; (**d**) cation exchange membrane.

In the salt-based alkaline electrolyte, samples’ open-circuit potentials, impedance curves, and potentiodynamic polarization curves were examined consecutively. Open-circuit potential curves were taken from 0 to 3600 s. Ultimate open-circuit potential was the alloy’s average stabilization phase potential. The open-circuit voltage scanned from −2 to 0 V at a rate of 1 mV/s. Assuming steady open-circuit voltage, the impedance curve was measured using a 5 mV sine wave with a frequency range of 10^−1^~10^5^ Hz, using ZSimpWin software (version 3.5, Echem Software, Ann Arbor, MI, USA) for data fitting. Polarization curve test voltage range was −2~0 V, with a scanning rate of 10 mV/s. Under steady open-circuit voltage, the impedance curve was measured using a 5 mV sine wave with a frequency range of 10^−1^~10^5^ Hz. The experimental mold and sample are shown in Figure 21 below.

The constant current discharge test method simulates the battery’s operation in a relatively ideal way by discharging the electrode under constant current, recording the battery’s voltage value as the test progresses, and calculating the electrode’s specific capacity, energy, study material electrochemistry, etc. The equations for anode use, capacity density, and energy density were as follows:

(2) $$\varpi =\frac{1000I\cdot t}{\Delta m\cdot F}$$

(3) $$Q=\frac{I\cdot h}{\Delta m}$$

(4) $$W=\frac{U\cdot I\cdot h}{\Delta m}$$

where ϖ was the anode utilization rate, %; *Q* was the volume density, mAh/g; *W* was the energy density, wAh/g; *I* was the discharge current, mA; *t* was the discharge time, s; Δm was the weight loss during discharge process, g; *F* was the Faraday constant, 96,485 C/mol; *h* was the discharge time, *h*; and *U* was the discharge voltage, V.

At the end of the test, the discharged samples were immersed in dilute nitric acid solution for 120 s. After removal, the samples were ultrasonically cleaned with anhydrous ethanol, dried with a hair dryer, and weighed. The surface morphology of the aluminum anode after discharge was observed and analyzed using a SU1510 scanning electron microscope (SEM) manufactured by Hitachi, Ltd. (Hitachi, Tokyo, Japan) in Japan.

## 4. Conclusions

In this work, Al6061/0.5wt.% composite anode was manufactured using SLM, and the laser power was changed to study its impact on the overall performance of the Al anode. The testing results revealed that 340 W was considered as a relative optimal laser power to print the Al6061/0.5wt.% composite anode. Detailed conclusions were given as follows.

The laser power has a great impact on the molten pool size, which resulted in the voids and cracks formation on the SLMed parts. When the laser power was too low, the formation of the molten pool temperature was low, which resulted in the reduction in Marangooni convection strength. This leads to the formation of voids. When the laser power was too high, excessive energy was input into the melt pool, overheating it, pushing the molten pool center to the edge of the very high temperature gradient caused by a strong inward Marangoni flow, exacerbating the instability of the melt flow, increasing gas capture, and making porosity defects easy to form.

With the increasing size and amount of voids and cracks, electrolytes were more easily leaked into the center of the anode, which resulted in the voids and cracks increasing on the surface of the sample and caused severe corrosion expansion to occur. The self-corrosion rate, open-circuit potential, polarization curve, EIS curve, and constant-current discharge performance all confirmed the results given above. The morphologies of the surface morphology of anodes after discharge further verified the conclusions given before.

## Figures and Tables

**Table 1:** Self-corrosion rate of Al composite anode printed under different laser powers.

Laser Power	Self-Corrosion Rate *v*_a_ (g·cm^−2^·min^−1^)
260 W	1.344 × 10^−4^
300 W	1.056 × 10^−4^
340 W	8.804 × 10^−5^
380 W	1.026 × 10^−4^
420 W	1.163 × 10^−4^

**Table 2:** Stable open-circuit potentials reached TiB_2_/6061 composite anode printed under different laser powers.

Laser power/W	260	300	340	380	420
OCP/V	−1.499	−1.527	−1.569	−1.537	−1.529

**Table 3:** Polarization curve fitting data of TiB_2_/6061 composites printed under different laser powers.

Laser Power	Polarization Curve Fitting Data		
	E_corr_, (V vs.Hg/HgO)	I_corr_, (A·cm^−2^)	R_p_, (Ω∙cm^2^)
260 W	−1.520	5.014 × 10^−3^	9.2
300 W	−1.530	4.823 × 10^−3^	10.1
340 W	−1.544	3.819 × 10^−3^	12.4
380 W	−1.536	4.467 × 10^−3^	11.2
420 W	−1.519	4.692 × 10^−3^	10.3

**Table 4:** Fitting parameters of electrochemical impedance spectra of anodes printed at different laser powers.

	260 W	300 W	340 W	380 W	420 W
L/H·cm^2^	7.270 × 10^−7^	9.079 × 10^−7^	7.316 × 10^−7^	1.150 × 10^−6^	7.596 × 10^−7^
R_s_/Ω·cm^2^	2.07	1.706	2.152	2.304	2.051
CPE_1_/F·cm^−2^	2.475 × 10^−4^	4.867 × 10^−4^	2.506 × 10^−4^	3.471 × 10^−4^	2.550 × 10^−4^
R_t_/Ω·cm^2^	2.827	4.352	8.504	4.125	3.235
CPE_2_/F·cm^−2^	6.822 × 10^−2^	3.640 × 10^−2^	2.647 × 10^−2^	6.281 × 10^−2^	5.624 × 10^−2^
R_c_/Ω·cm^2^	2.147	3.76	8.348	3.505	2.4
χ^2^	2.783 × 10^−4^	4.758 × 10^−4^	1.960 × 10^−4^	5.132 × 10^−4^	2.012 × 10^−4^

**Table 5:** Discharge parameters of TiB_2_/6061 composites anode printed under different laser powers.

Laser Power /W	Average Discharge Voltage/V	Specific Capacity/(mAh·g^−1^)	Energy Density/(Wh·kg^−1^)	Anode Usage Rate/%
260	−1.153 ± 0.002	1436.15	1656.52	48.25
300	−1.187 ± 0.003	1507.24	1792.68	50.71
340	−1.254 ± 0.003	1612.58	2022.21	54.16
380	−1.222 ± 0.004	1534.17	1875.23	51.53
420	−1.174 ± 0.004	1470.59	1727.12	49.39

**Table 6:** Chemical composition of 6061 powder.

Element	Si	Cu	Mg	Cr	Mn	Fe	Zn	Al
wt.%	0.55	0.24	1.13	0.16	<0.03	<0.70	0.035	Bal.

**Table 7:** Parameters of SLM machine used in this work.

Model	XDM250
Size	1670 mm × 1140 mm × 2160 mm
Forming space	250 mm × 250 mm × 410 mm
Layer thickness	Minimum 20 μm
Laser type	IPG fiber laser 500 W
Wavelength	1070 nm
Hardware	XDM IntelliProc^®^, XDM IntelliMake^®^
Powder supply	Lower powder supply

**Table 8:** Single-factor experiment.

Laser Power/W	Scan Speed/mm·s^−1^	Scam Distance/μm
260, 300, 340, 380, 420	1000	140

## Data Availability

All the data are shown in manuscript.

## References

[1] Arroyo F., Miguel L.J. (2019). Analysis of energy demand scenarios in Ecuador: National government policy perspectives and global trend to reduce CO_2_ emissions. Int. J. Energy Econ. Policy.

[2] De Cian E., Sue Wing I. (2019). Global energy consumption in a warming climate. Environ. Resour. Econ..

[3] Egan D.R., De León C.P., Wood R.J.K., Jones R.L., Stokes K.R., Walsh F.C. (2013). Developments in electrode materials and electrolytes for aluminium–air batteries. J. Power Sources.

[4] Han Y.-F., Dai Y.-B., Shu D., Wang J., Sun B.-D. (2006). First-principles calculations on the stability of Al/TiB_2_ interface. Appl. Phys. Lett..

[5] Xi L.X., Wang P., Prashanth K.G., Li H., Prykhodko H.V., Scudino S., Kaban I. (2019). Effect of TiB2 particles on microstructure and crystallographic texture of Al−12Si fabricated by selective laser melting. J. Alloys Compd..

[6] Wang P., Gammer C., Brenne F., Niendorf T., Eckert J., Scudino S. (2018). A heat treatable TiB2/Al−3.5Cu−1.5Mg−1Si composite fabricated by selective laser melting:Microstructure, heat treatment and mechanical properties. Compos. Part B Eng..

[7] Cao Z., Li J.L., Zhang H.P., Li W.B., Wang X.D. (2020). Mechanical and tribological properties of graphene nanoplatelets-reinforced titanium composites fabricated by powder metallurgy. J. Iron Steel Res. Int..

[8] Gu D.D., Wang H.Q., Zhang G.Q. (2014). Selective laser melting additive manufacturing of Ti-based nanocomposites: The role of nanopowder. Metall. Mater. Trans. A.

[9] Liu J., Gu D., Chen H., Dai D., Zhang H. (2018). Influence of surface topography on orbital wetting behavior of selected zone laser melted aluminum alloy substrates. J. Zhejiang Univ. Sci. A.

[10] Prashanth K.G., Scudino S., Eckert J. (2017). Defining the tensile properties of Al-12Si parts produced by selective laser melting. Acta Mater..

[11] Li W., Li S., Liu J., Zhang A., Zhou Y., Wei Q., Yan C., Shi Y. (2016). Effect of heat treatment on AlSi10Mg alloy fabricated by selective laser melting: Microstructure evolution, mechanical properties and fracture mechanism. Mater. Sci. Eng. A.

[12] Akram J., Chalavadi P., Pal D., Stucker B. (2018). Understanding grain evolution in additive manufacturing through modeling. Addit. Manuf..

[13] Uddin S.Z., Murr L.E., Terrazas C.A., Morton P., Roberson D.A., Wicker R.B. (2018). Processing and characterization of crack-free aluminum 6061 using high-temperature heating in laser powder bed fusion additive manufacturing. Addit. Manuf..

[14] Martin J.H., Yahata B.D., Hundley J.M., Mayer J.A., Schaedler T.A., Pollock T.M. (2017). 3D printing of high-strength aluminium alloys. Nature.

[15] Xi L., Gu D., Guo S., Wang R., Ding K., Prashanth K.G. (2020). Grain refinement in laser manufactured Al-based composites with TiB_2_ ceramic. J. Mater. Res. Technol..

